# De novo POGZ mutations in sporadic autism disrupt the DNA-binding activity of POGZ

**DOI:** 10.1186/s40303-016-0016-x

**Published:** 2016-04-21

**Authors:** Kensuke Matsumura, Takanobu Nakazawa, Kazuki Nagayasu, Nanaka Gotoda-Nishimura, Atsushi Kasai, Atsuko Hayata-Takano, Norihito Shintani, Hidenaga Yamamori, Yuka Yasuda, Ryota Hashimoto, Hitoshi Hashimoto

**Affiliations:** Laboratory of Molecular Neuropharmacology, Graduate School of Pharmaceutical Sciences, Osaka University, 1-6 Yamadaoka, Suita, Osaka 565-0871 Japan; iPS Cell-Based Research Project on Brain Neuropharmacology and Toxicology, Graduate School of Pharmaceutical Sciences, Osaka University, 1-6 Yamadaoka, Suita, Osaka 565-0871 Japan; Department of Psychiatry, Osaka University Graduate School of Medicine, D3, 2-2, Yamadaoka, Suita, Osaka 565-0871 Japan; Molecular Research Center for Children’s Mental Development, United Graduate School of Child Development, Osaka University, Kanazawa University, Hamamatsu University School of Medicine, Chiba University and University of Fukui, 2-2, Yamadaoka, Suita, Osaka 565-0871 Japan

**Keywords:** Autism spectrum disorder, Recurrent mutation, De novo mutation, POGZ, DNA-binding activity

## Abstract

**Background:**

A spontaneous de novo mutation is a new mutation appeared in a child that neither the parent carries. Recent studies suggest that recurrent de novo loss-of-function mutations identified in patients with sporadic autism spectrum disorder (ASD) play a key role in the etiology of the disorder. POGZ is one of the most recurrently mutated genes in ASD patients. Our laboratory and other groups have recently found that POGZ has at least 18 independent de novo possible loss-of-function mutations. Despite the apparent importance, these mutations have never previously been assessed via functional analysis.

**Methods:**

Using wild-type, the Q1042R-mutated, and R1008X-mutated POGZ, we performed DNA-binding experiments for proteins that used the CENP-B box sequence in vitro. Data were statistically analyzed by one-way ANOVA followed by Tukey-Kramer post hoc tests.

**Results:**

This study reveals that ASD-associated de novo mutations (Q1042R and R1008X) in the POGZ disrupt its DNA-binding activity.

**Conclusions:**

Here, we report the first functional characterization of de novo POGZ mutations identified in sporadic ASD cases. These findings provide important insights into the cellular basis of ASD.

## Background

**Fig. 1:**
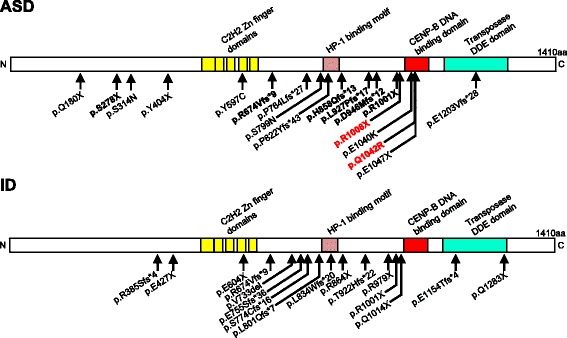
Schematic structure of POGZ and its putative functional domains. The ASD-(upper) and ID- (lower) associated de novo mutations are indicated below the protein. Bold mutations, common de novo mutations between ASD and ID. Note that the R1001X mutation was found in ID and ASD/ID patients. X, nonsense; del, deletion; fs, frameshift; *, premature stop codon

The genetic etiology of autism spectrum disorder (ASD) remains poorly understood. A spontaneous de novo mutation is a new mutation appeared in a child that neither the parent carries. Recent next-generation sequencing studies have demonstrated that de novo mutations greatly contribute to the risk of ASD and often produce large effects [1–5]. In particular, genes with highly recurrent de novo possible loss-of-function mutations play key roles in the etiology of this disorder. De novo mutations in multiple (≥3) unrelated patients have been identified in several such high-confidence ASD risk genes, including *CHD8*, *ARID1B*, *SYNGAP1*, *DYRK1A*, *SCN2A*, *ANK2*, *ADNP*, *DSCAM*, *CHD2*, *KDM5B*, *SUV420H1*, *GRIN2B*, *ASH1L*, and POGZ [5]. Among these 14 genes, POGZ is one of the most recurrently mutated genes in ASD patients [4, 5]. Our laboratory and other groups have recently found that POGZ has at least 18 independent de novo possible loss-of-function mutations (Fig. 1, upper) [4–8]. Therefore, de novo mutations in POGZ can be strongly associated with ASD risk; however this association requires experimental validation. Despite the apparent importance, these mutations have never previously been assessed via functional analysis. Here, we report that ASD-associated de novo mutations in the POGZ disrupt the DNA-binding activity of POGZ. These findings provide insight into the cellular basis of ASD. In addition, de novo POGZ mutations are frequently found also in patients with intellectual disability (ID) [7, 9–11] (Fig. 1, lower). Our current findings may also help to understand the molecular etiology of ID.

## Methods

### Cell culture and DNA transfection

Human POGZ cDNA was purchased from DNAFORM (clone ID: 30745658, Kanagawa, Japan), amplified via PCR, and subcloned into a pcDNA-6Myc expression vector. Single amino acid mutants of POGZ were generated using a KOD mutagenesis kit (Toyobo, Osaka, Japan) in accordance with the manufacturer’s instructions. HEK293T cells were cultured in Dulbecco’s Modified Eagle Medium supplemented with 10 % fetal bovine serum and were transfected using TransIT Transfection Reagent (Takara, Ohtsu, Japan). Two days later, cells were harvested and lysed with TNE buffer (50 mM Tris–HCl (pH 7.5); 100 mM NaCl; 5 mM EDTA; 0.1 % (w/v) NP-40) for the DNA-binding assay [12].

### Antibodies

Antibodies used in this study were obtained commercially and included antibodies against POGZ (Sigma-Aldrich, MO, USA), Tuj1 (Covance, CA, USA), GAPDH (Millipore, MA, USA), Histone H3 (Cell Signaling, MA, USA), and Myc (9E10) (Santa Cruz, CA, USA).

### Cortical neuronal cultures and neuron immunocytochemistry

Cortical cultures were prepared from E16.5 embryonic mouse cortex in minimum essential medium with B27 supplement and 5 % fetal bovine serum and plated on glass coverslips coated with poly-L-lysine, as previously described [13]. Neuron immunocytochemistry (at 7 days in vitro) was performed as previously described [13].

### Preparation of cytosolic and nuclear fractions

The preparation of cytosolic and nuclear fractions from dissociated cortical neurons (at 7 days in vitro) was performed using a Cytoplasmic & Nuclear Protein Extraction Kit (101Bio, CA, USA) in accordance with the manufacturer’s instructions.

### Immunoblotting

Bead-bound proteins and lysates were resolved by SDS-PAGE and transferred to polyvinylidene difluoride membranes. Subsequently, these membranes were probed with the indicated antibodies. Data acquisition and analysis were performed using an LAS 4000 image analyzer (GE Healthcare, NJ, USA).

### Assay for POGZ-CENPB-DB domain binding

HEK293T cell lysates expressing virtually equivalent levels of Myc-tagged wild-type or mutant POGZ were prepared. These lysates were mixed with 10 μg of a 3’-biotinylated DNA fragment carrying the CENP-B box sequence [14] and with NeutrAvidin beads (30 μl, 50 % slurry, Thermo Scientific, MA, USA) for 4 h at 4 °C. After incubation, the beads were collected and washed 4 times with TNE buffer. For quantification, the precipitated POGZs were normalized to each POGZ level in total lysates. The background level was set to the band intensity for the precipitated R1008X POGZ mutant. The levels of precipitated POGZs were normalized to each POGZ level in total lysates. Data were statistically analyzed by one-way ANOVA followed by Tukey-Kramer post hoc tests. Differences were considered significant if *p* < 0.05.

## Results

**Fig. 2:**
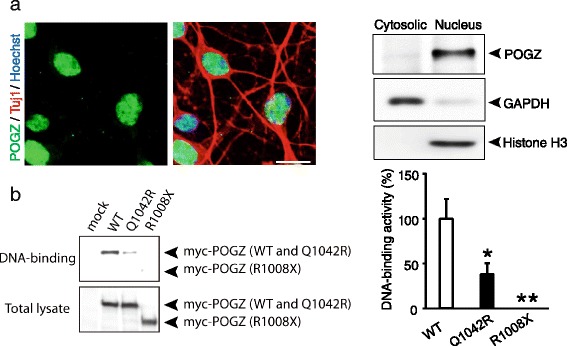
ASD-associated de novo R1008X and Q1042R mutations disrupt the DNA-binding activity of POGZ. **a** POGZ is localized to the nucleus in dissociated embryonic cortical neurons (7 days in vitro). left, Double immunostaining for POGZ (green) and a neuron marker, Tuj1 (red). Nuclei were stained with Hoechst 33258 (blue). Scale bar, 10 μm. right, Biochemical preparation of the cytosolic and nuclear fractions from dissociated neurons. Equal quantities of protein were loaded into individual lanes and probed with antibodies against POGZ, GAPDH (a cytosolic marker), and Histone H3 (a nuclear marker). **b** Wild-type (WT) but not R1008X POGZ binds a CENP-B box sequence. left, Representative western blots. right, Quantification of DNA-binding activity. Notably, the Q1042R mutation led to a reduction of approximately 60 % in DNA binding (each *n* = 6, *p < 0.05, **p < 0.01, one-way ANOVA followed by Tukey-Kramer post hoc tests (vs. WT)). The averaged WT value was set to 100 %. Data are expressed as the means ± SEM

POGZ encodes a heterochromatin protein 1α-binding protein that contains a zinc-finger cluster, an HP1-binding motif, a centromere protein-B-like DNA-binding (CENPB-DB) domain, and a transposase-derived DDE domain [15]. Thus, it has been suggested that POGZ functions as a chromatin regulator [15]. In accordance with this hypothesis, we found that POGZ was localized to the nucleus in neurons (Fig. 2a); however, the function of POGZ in the central nervous system is unknown. We recently discovered a Q1042R amino acid substitution within the CENPB-DB domain in sporadic ASD cases (Fig. 1, upper) [8]. Given that the CENPB-DB domain is likely to be involved in CENP-B box sequence-specific DNA-binding [14], this substitution may affect the DNA-binding activity of POGZ. To examine this possibility, we performed DNA-binding experiments for proteins that used the CENP-B box sequence (Fig. 2b) [14]. We prepared HEK293T cell lysates expressing virtually equivalent levels of Myc-tagged wild-type, Q1042R-mutated, and R1008X-mutated POGZ (Fig. 2b, left, lower). These lysates were mixed with the DNA fragment carrying the CENP-B box sequence. The levels of precipitated POGZs were normalized to each POGZ level in total lysates (Fig. 2b, right). We found that wild-type POGZ co-precipitated well with the DNA fragment carrying the CENP-B box sequence, a clear indication of binding between wild-type POGZ and the CENP-B box sequence (Fig. 2b). Interestingly, the Q1042R mutation was associated with a reduction of approximately 60 % in DNA-binding, suggesting that Q1042 is important for the DNA-binding activity of POGZ (Fig. 2b). We also examined the DNA-binding activity of POGZ carrying the ASD-associated R1008X de novo mutation; this mutation results in a truncated protein that lacks the entire CENPB-DB domain. We found that POGZ with this mutation did not co-precipitate with the DNA fragment (Fig. 2b), indicating the importance of the CENPB-DB domain to the DNA-binding activity of POGZ.

## Discussion

Recently, POGZ is found to be important for normal learning in a habituation paradigm in *Drosophila* [7]; however, the significance of disease-associated de novo mutations remains unclear. Here, we report the first functional characterization of de novo POGZ mutations identified in sporadic ASD cases. Our results indicate that ASD-associated de novo mutations disrupt the DNA-binding activity of POGZ, an effect likely to result in the perturbation of chromatin function and the neuronal transcription network. Given that chromatin regulation plays an essential role in gene expression and cellular function, the disruption of chromatin-related mechanisms causes pathological effects on brain function [2, 16]. Importantly, 8 out of 14 recurrently mutated high-confidence ASD risk gene products, including POGZ, ADNP, ARID1B, CHD2, KDM5B, SUV420H1, ASH1L, and CHD8, are likely to be chromatin regulators [5]; this finding indicates the critical involvement of chromatin regulation and function in the etiology of ASD [2, 16]. Interestingly, it has been suggested that POGZ cooperatively regulates chromatin structure and gene expression during human neurodevelopment in combination with a chromatin modifier CHD8, which harbors the largest number of loss-of-function mutations in sporadic ASD [5, 17–19]. Taken together, these findings indicate that both POGZ and *CHD8* may be strongly associated with ASD risk; however the way in which these disruptive de novo mutations of chromatin regulators are involved in ASD risk remains unclear [17–19]. It is important to identify the transcriptional targets of POGZ and CHD8 as well as the biological significance of the disruptive de novo mutations.

Many genes are shown to be associated with ID as well as ASD. Recent studies show that candidate ID- and ASD-associated genes are likely to be largely overlapping [20]. As expected, in addition to ASD [4–8], de novo POGZ mutations are frequently found also in patients with intellectual disability [7, 9–11]. Patients with POGZ mutations show borderline-moderate ID [7] and lower IQ score [8]. Therefore, identification of the significance of disease-associated de novo mutations in the POGZ may unravel the common neural systems associated with ASD and ID.

## Conclusions

Our current results, which indicate that de novo mutations in POGZ impair the DNA-binding activity of POGZ, significantly contribute to understanding the molecular link between chromatin remodeling and ASD. Further analysis of the function of de novo mutations in chromatin regulators will provide important clues to the molecular pathophysiology of ASD.

## Abbreviations

ASD: autism spectrum disorder
CENPB-DB: centromere protein-B-like DNA binding
